# Purinergic receptors mediate endothelial dysfunction and participate in atherosclerosis

**DOI:** 10.1007/s11302-021-09839-x

**Published:** 2022-01-03

**Authors:** Xian-Ming Wu, Ning Zhang, Jiang-Shan Li, Zhi-Hong Yang, Xiao-Lou Huang, Xiao-Fang Yang

**Affiliations:** 1 Hunan University of Chinese Medicine, Changsha, 410208 China; 2 Guizhou University of Traditional Chinese Medicine, Guiyang, 550025 China

**Keywords:** Atherosclerosis, Purinergic signaling, Endothelial dysfunction, P1 receptors, P2 receptors

## Abstract

Atherosclerosis is the main pathological basis of cardiovascular disease and involves damage to vascular endothelial cells (ECs) that results in endothelial dysfunction (ED). The vascular endothelium is the key to maintaining blood vessel health and homeostasis. ED is a complex pathological process involving inflammation, shear stress, vascular tone, adhesion of leukocytes to ECs, and platelet aggregation. The activation of P2X4, P2X7, and P2Y2 receptors regulates vascular tone in response to shear stress, while activation of the A2A, P2X4, P2X7, P2Y1, P2Y2, P2Y6, and P2Y12 receptors promotes the secretion of inflammatory cytokines. Finally, P2X1, P2Y1, and P2Y12 receptor activation regulates platelet activity. These purinergic receptors mediate ED and participate in atherosclerosis. In short, P2X4, P2X7, P2Y1, and P2Y12 receptors are potential therapeutic targets for atherosclerosis.

## Introduction

Atherosclerosis is a chronic vascular disease and is the main pathological basis of cardiovascular disease (CVD) [1, 2]. Globally, CVD caused by atherosclerosis is spreading [3], leading to a heavy worldwide disease and economic burden [4]. Its pathogenesis involves lipid infiltration, endothelial injury, inflammation, and plaque formation [4], with endothelial injury and an abnormal inflammatory response being the keys to its occurrence [5]. As the physical barrier between the blood and blood vessel wall, the vascular endothelium is essential for maintaining blood vessel homeostasis [6]. Endothelial cells (ECs) bridge the blood and blood vessel walls and play a critical role in cardiovascular homeostasis by regulating shear stress, vascular tone, monocyte or leukocyte adhesion, and platelet aggregation [7]. Healthy ECs regulate the secretion and balance of vasodilating, vasoconstricting, anti-inflammatory, pro-inflammatory, oxidative, and antioxidant factors. A properly functioning vascular endothelium is regarded as the gatekeeper to cardiovascular health [8]. ECs are the first barrier in protecting blood vessels. Accordingly, atherosclerosis begins with their injury and dysfunction, which leads to plasma lipids invading the endothelium, monocyte or macrophage infiltration, vascular smooth muscle cell migration to the intima, and the formation of foam cells via the engulfment of lipids by macrophage. Moreover, ECs secrete a variety of pro-inflammatory factors, which eventually evolve into atherosclerosis [9].

A normally functioning endothelium regulates vascular tone by balancing the secretion of vasodilators and vasoconstrictors [10]. The decreased expression of vasodilatory factors such as nitric oxide (NO), prostacyclin I_2_ (PGI_2_), or endothelial-derived hyperpolarizing factor (EDHF) or the increased expression of vasoconstrictor factors such as endothelin 1 (ET-1), angiotensin II, or thromboxane A_2_ (TxA_2_) will imbalance vascular homeostasis and cause ED. As a semipermeable membrane, the vascular endothelium forms a biological barrier between the intravascular and extravascular spaces to regulate the transportation and exchange of molecules [11]. An incomplete endothelial barrier leads to vascular hyperpermeability, vascular swelling and edema, and eventually EC damage. ED is key in the occurrence and development of cardiovascular and cerebrovascular diseases. The main causes of ED are lipid metabolism disorders, inflammation, oxidative stress, and shear stress [12]. Under the action of these factors, ECs reduce the synthesis or activity of NO, PGI2, EDHF, and other vasodilators and increase the synthesis of vasoconstrictors such as endothelin (ET). Consequently, this perturbation of the balance between vasodilation and vasoconstriction induces the production of reactive oxygen species (ROS) and the release of the pro-inflammatory cytokines interleukin-1 (IL-1), interleukin-6 (IL-6), and tumor necrosis factor-α. Furthermore, the bioavailability of NO is reduced, which ultimately leads to ED [13].

Endothelial injury causes the abnormal expression of intercellular adhesion molecule-1 (ICAM-1), vascular cell adhesion molecule-1 (VCAM-1), and platelet-endothelial cell adhesion molecule-1 (PECAM-1); it also promotes the adhesion of leukocytes to ECs. ICAM-1, VCAM-1, and PECAM-1 are specific indicators of vascular endothelial injury. Shear stress acts on ECs, causing leukocytes to migrate to adhesion molecules and chemokines in the arterial wall, leading to atherosclerosis [14]. The evaluation of EC function is an important tool for predicting the occurrence of CVD [15]. The improvement of EC function reduces the occurrence of CVD events, which include atherosclerosis [16, 17]. Interestingly, purinergic signaling is critical in regulating vascular tone and remodeling [18]. Increasing studies have reported that purinergic signaling mediates ED and participates in the formation of atherosclerosis [19].

In 1972, Burnstock introduced the concept of purinergic signaling, whereby he identified adenosine triphosphate (ATP) as an extracellular signaling molecule and called it a purinergic neurotransmitter [20]. In 1976, he suggested that extracellular purinergic signaling acts via purinergic receptors, thus first proposing the purinergic receptors [21]. The purinergic signaling system includes purinergic substances and their receptors. These purinergic substances include ATP, adenosine diphosphate (ADP), adenosine monophosphate (AMP), and adenosine. Almost all cells release ATP; it is the main universal energy currency in the cell, and it is released outside the cell as a signal molecule [22]. Intracellularly, ATP, ADP, and AMP are converted into adenosine via cytoplasmic 5′-nucleosidase activity. ATP can also be converted to cyclic adenosine monophosphate (cAMP) by adenylyl cyclase, which is then subsequently converted to AMP by phosphodiesterase. Cytoplasmic 5′-nucleotidase then converts AMP to adenosine. Additionally, adenosine is converted into inosine by adenosine deaminase, and adenosine can be converted back into AMP by adenosine kinase [23]. Extracellularly, ATP is released to the extracellular space via transporter-mediated or ATP-permeable ion channels, such as the pannexin 1 channel [24]. ATP is hydrolyzed to ADP and AMP mainly via the continuous action of ectonucleoside diphosphohydrolase triphosphate (CD39), while ecto-5′-nucleotidase (CD73) converts the AMP produced by the ATP and cAMP pathway into adenosine [25].

Purinergic receptors can be subdivided into two categories: P1 and P2. P1 receptors are G protein-coupled and consist of the A1, A2A, A2B, and A3 receptor subtypes [26]; they are also known as adenosine receptors [27]. P2 receptors consist of two types, P2X and P2Y. P2X are ligand-gated cationic channel receptors and can be subdivided into the P2X1-7 receptor subtypes [28]. They mediate rapid responses to ATP [29]. By contrast, P2Y receptors are G protein-coupled and can be divided into the P2Y1, P2Y2, P2Y4, P2Y6, and P2Y11-14 receptor subtypes [28]; they respond to ATP and ADP [29]. Notably, the role of purinergic substances and their receptors in physiological and pathological processes has recently received more attention [30–32].

## Purinergic receptors and endothelial dysfunction

Purinergic receptors are distributed throughout most tissues or cells and are the basis of endothelial-mediated vasodilation [33], inflammation [34], cell differentiation, migration, and proliferation [35]. Adenosine and ATP mediate the migration, growth, and proliferation of ECs [35]. During vascular remodeling, ATP and adenosine act on ECs via P1 and P2Y receptors to promote proliferation [19]. The purinergic activation of ECs leads to the release of NO, PGI2, and EDHF, thereby causing vasodilation [36, 37]. More specifically, endothelial P2Y receptors mediate vasodilation by releasing NO and EDHF [33].

**Fig. 1:**
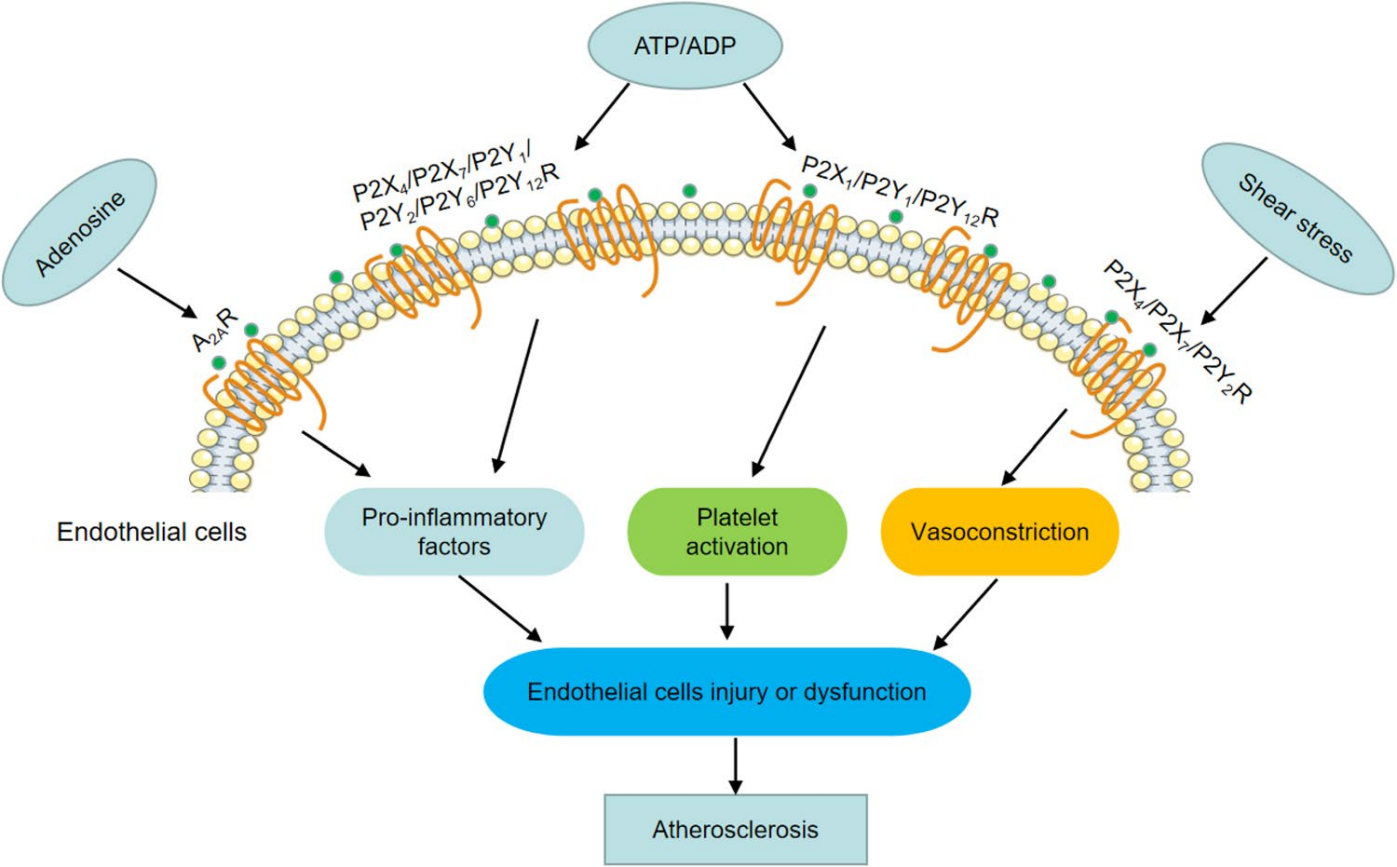
Purinergic receptors mediate ED and participate in atherosclerosis. A2A, A2B, A3, P2X1, P2X4, P2X7, P2Y1, P2Y2, P2Y6, P2Y11, and P2Y12 receptors mediate endothelial cell dysfunction and participate in atherosclerosis. The activation of P2X4, P2X7, and P2Y2 receptors regulates vascular tone in response to shear stress. The activation of A2A, P2X4, P2X7, P2Y1, P2Y2, P2Y6, and P2Y12 receptors promotes the secretion of inflammatory cytokine interleukin (IL)-1β, 1L-6, IL-8, IL-1, intercellular adhesion molecule-1 and vascular cell adhesion molecule-1. P2X1, P2Y1, and P2Y12 receptor activation regulates platelet activity (platelet aggregation), mediates ED, and participates in atherosclerosis. R, receptors

Reductions in NO bioavailability and increases in ROS release can cause ED [38]. Damage to the barrier function of the vascular endothelium promotes the deposition of low-density lipoprotein under the vascular intima, which is then modified into oxidized low-density lipoprotein (ox-LDL). As a key molecule in atherosclerosis, ox-LDL can bind to receptors and trigger a series of intracellular changes that cause vascular EC injury and dysfunction. The stimulation of monocyte macrophages to excessively engulf ox-LDL accelerates the transformation of macrophages into foam cells and forms plaques under the vascular endothelium, thereby promoting atherosclerosis development [39]. Notably, ox-LDL stimulates ECs to release ATP, thereby activating P2Y2 receptor. On one hand, it increases caspase-1 activity and interleukin-1β (IL-1β) secretion and activates the inflammasome [40]. On the other hand, it promotes ROS release, increases ICAM-1 and VCAM-1 content, promotes monocyte migration and EC adhesion, and participates in the pathogenesis of atherosclerosis [34]. P2Y2 receptors are an important medium for ox-LDL-mediated monocyte adhesion to ECs [34]. Table 1 summarizes the ED caused by purinergic receptors activation or inhibition, and Fig. 1 summarizes the role of purinergic receptors in atherosclerosis via their regulation of vascular tone, inflammation, and platelet aggregation.

**Table 1:** Purinergic receptor activation or inhibition leads to endothelial dysfunction

	P1	P2X	P2Y
Increased vascular tone		P2X4 (-)	P2Y2 (-)
Inflammation		P2X7 (-)	
	A2A (-)	P2X4 ( +)	P2Y1 ( +)
	A1 ( +)	P2X7 ( +)	P2Y2 ( +)
			P2Y6 ( +)
			P2Y12 ( +)
Platelet activation		P2X1 ( +)	P2Y1 ( +)
			P2Y12 ( +)

+ , activation; − , inhibition

Activation or inhibition of different purinergic receptors leads to endothelial dysfunction by regulating vascular tone, inflammation, and platelet activity, thereby indicating their participation in atherosclerosis

## P1 receptors in endothelial dysfunction

A1, A2A, A2B, and A3 receptors are expressed on ECs [41], among which the A2A and A2B receptors are the main purinergic receptors on ECs [42]. A2A receptor knockout in apolipoprotein E-deficient mice prevents the formation of atherosclerotic lesions [43]. Under hypoxic conditions, adenosine activates A3 and A2B receptors, increases vascular endothelial growth factor (VEGF) secretion, and promotes foam cell formation [44]. A2A and A2B receptors activation will increase cAMP levels, leading to vasodilation [45]. The subcutaneous injection of A2A receptor agonist in mice decreases VCAM-1, ICAM-1, and P-selectin expression. Adenosine inhibits early inflammation by activating the A2A receptors of ECs [46]. The knockout or inhibition of A1 receptor also reduces the concentration of pro-inflammatory cytokines, thereby reducing atherosclerotic lesions in apolipoprotein E-deficient mice [47]. In short, P1 receptors mediate ED and participate in atherosclerosis.

## P2 receptors in endothelial dysfunction

P2X4, P2X7, P2Y1, P2Y2, P2Y6, and P2Y11 receptors are the most highly expressed P2 receptors in ECs and may be related to the release of ATP, NO, and EDHF [48]. P2X receptors are also expressed on ECs and are related to cell adhesion and permeability. The stimulation of human ECs via P2Y1 receptors activate VEGF-2 and leads to angiogenesis [49]. P2Y2 receptors in ECs regulate angiopoietin-2 and VEGFR-2 expression and play a crucial role in angiogenesis [50]. Endothelial P2Y2 receptor knockout will reduce endothelial nitric oxide synthase (eNOS) activity, resulting in reduced NO production, causing vasoconstriction, and ultimately leading to ED [51]. Ticagrelor, a P2Y12 inhibitor, improves endothelial function by activating endothelial eNOS in the vascular endothelium, thereby reducing circulating epidermal growth factor [52].

Shear stress is the frictional resistance acting on the vascular cavity surface [53]. It can regulate the structure and function of the endothelium [54, 55] and is closely related to the development, physiology, and pathology of the blood vessel. ECs transduce fluid shear stress into biochemical signals to regulate endothelial function [56]. That is, in response to shear stress or hypoxia, ECs release ATP, which then act on P2X and P2Y receptors on the endothelium to release NO, PGI2, and EDHF, causing vasodilation [19, 57]. When the endothelium is injured, it promotes the release of vasoconstrictors, resulting in vasospasm [58]. Ca^2+^ plays an important role in the EC response to shear stress. As the P2X4 receptors of ECs participate in shear stress-mediated Ca^2+^ transmission, they may have shear sensor properties [59]. The ECs of mice with knockout P2X4 receptors respond to shear stress, and as the amount of released NO decreases, vascular tone increases [60]. Under the action of shear stress, ATP acts on ECs via P2X7 receptors, which induces E-selectin and IL-1β secretion and promotes endothelial inflammation in atherosclerotic sites [61]. In response to shear stress, ECs also release ATP, which acts on the P2Y2 receptor of ECs to induce NO release, and regulate vascular tone [62]. Correspondingly, deletion of the P2Y2 gene reduces shear stress-induced vasodilation [63].

Purinergic signaling mediating ED is involved in atherosclerosis, mainly via P2 receptor-mediated EC inflammation. ATP is released into the extracellular space and binds to P2 receptors to mediate inflammation [64]. During inflammation, ECs release ATP via the pannexin 1 channels to promote leukocyte adhesion and migration [24]. Notably, the release of ATP also involves the connexin 43 channels [65]. High glucose and palmitate upregulated the expression of P2X4 and P2X7 in human umbilical vein ECs, as well as increased the release of the inflammatory factors IL-1β, 1L-6, IL-8, ICAM-1, and VCAM-1. These results indicate that P2X4 and P2X7 receptors regulate high-glycemic inflammation in ECs. Hyperglycemia and hyperlipidemia cause ED, leading to oxidative stress and inflammation [66]. ATP acts on the P2X7 receptor, activates the inflammasome, and induces the release of inflammatory cytokines IL-1β and IL-18 as well as the production of ROS [67]. It promotes inflammation and ED and ultimately leads to atherosclerosis. Vascular traction injury has been reported to promote ATP release, which inhibits the production of NO via EC P2X7 receptors, leading to ED [68]. P2X7 receptors are highly expressed in mice with atherosclerotic lesions, and the realization of clinical patients is also consistent. P2X7-deficient mice exhibit reduced inflammasome activation and reduced leukocyte rolling and adhesion. These results indicate that P2X7 underactivity reduces atherosclerotic lesions by inhibiting the inflammatory response [69]. P2X7 receptor antagonists reduce IL-1β levels in atherosclerotic blood vessels [70]. As a ligand-gated ion channel, P2X7 receptor binds to extracellular ATP and promotes IL-1β release via the pannexin-1 channel [71].

P2Y1 receptor knockout in apolipoprotein E-deficient mice decreases macrophage infiltration and VCAM-1 levels, indicating that atherosclerosis is related to the P2Y1 receptors on ECs [72]. P2Y2 receptor causes vascular inflammation by increasing VCAM-1 expression in ECs, which eventually causes atherosclerosis [51]. In P2Y2 receptor-deficient mice, ATP-induced leukocyte adhesion is reduced. In addition, VCAM-1 and ICAM-1 RNA levels are decreased, indicating that extracellular ATP induces vascular inflammation and atherosclerosis by activating P2Y2 receptor [73]. In P2Y6-deficient mice, leukocyte adhesion to the vessel wall is reduced, as are ox-LDL and lipid content and RNA expression of ICAM-1 and IL-6. These reductions indicate that P2Y6 deficiency reduces inflammation and inhibits atherosclerosis [74]. During lipopolysaccharide-induced vascular inflammation, P2Y6 receptor knockout or injection of P2Y6 antagonists weakens the inflammatory response, indicating that P2Y6 receptor are closely related to vascular inflammation [75]. Inhibition P2Y12 receptor expression protects EC barrier function by increasing the concentration of cAMP in ECs [76]. Notably, P2Y11 receptor is also closely related to inflammation, as their stimulation reduces oxidative stress and improves ED [77].

Interestingly, purinergic signaling is also involved in atherosclerosis by regulating platelet activity. Platelets adhere and fuse into the vascular endothelium, thereby maintaining its integrity. P2X1, P2Y1, and P2Y12 receptors are expressed on platelets and mediate their aggregation [58]. ADP is a platelet agonist; it causes platelet shape change and aggregation via the P2X1, P2Y1, and P2Y12 receptors and generates TxA_2_ [78]. ATP diphosphohydrolase hydrolyzes extracellular ATP and ADP to AMP, and ADP inhibits platelet aggregation via ATP diphosphohydrolase. ATP diphosphohydrolase has the same functionality as CD39 [79]. CD39 is expressed on the surface of ECs, inhibits ADP-induced platelet aggregation, and is the main regulator of platelet activation [80]. Moreover, it protects against atherosclerosis [81]. Aspirin or clopidogrel, P2Y12 receptor antagonists that mediate platelet aggregation, is widely used to treat stroke and thrombosis [82, 83].

## Conclusion

Although a review of purinergic signaling in the cardiovascular system has been previously published [58], this review focuses on purinergic signaling mediating ED caused by atherosclerosis. Atherosclerosis begins with vascular ED, and ED involves purinergic signaling. ATP and its degradation products ADP, AMP, adenosine, uridine diphosphate, and cAMP stimulate a series of receptors and affect endothelial function. So far, the A2A, A2B, A3, P2X1, P2X4, P2X7, P2Y1, P2Y2, P2Y6, P2Y11, and P2Y12 receptors have been demonstrated to mediate EC dysfunction and participate in atherosclerosis.

This review reports that purinergic signaling mediates EC dysfunction and participates in atherosclerosis in different ways. Purinergic signaling plays an important role in regulating endothelial structure and function in response to shear stress. ECs release ATP in response to shear stress, which acts on P2X4 [60], P2X7 [61], and P2Y2 receptors [62] to regulate vascular tone. Disturbances in vascular tone will result in ED. Purinergic signaling also plays an important role in the inflammatory response, particularly by activating the A2A [46], P2X4 [66], P2X7 [69], and P2Y1 receptors [73] to induce the release of inflammatory factors and to promote the adhesion of leukocytes to ECs. Purinergic signaling also activates the P2X1, P2Y1, and P2Y12 receptors [78], causing platelet shape changes and aggregation and mediating ED to contribute to atherosclerosis.

In combination with the current understanding of the pathogenesis underlying atherosclerosis caused by ED and the in-depth understanding of purinergic signaling, further study is needed to determine the specific P2 receptors that mediate ED. Since different purinergic receptor subtypes can mediate similar functional effects, it is challenging to selectively target specific receptor subtypes. From this review, P2X4, P2X7, P2Y1, and P2Y12 receptors may be potential therapeutic targets for atherosclerosis. While few studies have been published on the involvement of P1 receptor-mediated ED in atherosclerosis, we will pay more attention to the relationship between P1 receptors and ED in the future. Therefore, purinergic receptors may become potential therapeutic targets for atherosclerosis.

## Data Availability

Not applicable.

## Abbreviations

CVD: Cardiovascular disease
ECs: Endothelial cells
ED: Endothelial dysfunction
NO: Nitric oxide
PGI_2_: Prostacyclin I_2_
EDHF: Endothelium-derived hyperpolarizing factor
ET-1: Endothelin 1
TXA_2_: Thromboxane A_2_
ET: Endothelin
ROS: Reactive oxygen species
IL-1: Interleukin-1
IL-6: Interleukin-6
ICAM-1: Intercellular adhesion molecule-1
VCAM-1: Vascular cell adhesion molecule-1
PECAM-1: Platelet-endothelial cell adhesion molecule-1
ATP: Adenosine triphosphate
ADP: Adenosine diphosphate
AMP: Adenosine monophosphate
cAMP: Cyclic adenosine monophosphate
CD39: Ectonucleoside diphosphohydrolase triphosphate
CD73: Ecto-5′-nucleotidase
ox-LDL: Oxidized low-density lipoprotein
IL-1β: Interleukin-1β
VEGF: Vascular endothelial growth factor
eNOS: Endothelial nitric oxide synthase
